# A Visualization of a Socio-Ecological Model for Urban Public Mental Health Approaches

**DOI:** 10.3389/fpubh.2021.654011

**Published:** 2021-05-13

**Authors:** Antonis A. Kousoulis, Isabella Goldie

**Affiliations:** Mental Health Foundation, London, United Kingdom

**Keywords:** mental health, social determinansts of health, public health, socio – ecological system, urban health

## Introduction

According to the latest data from the Global Burden of Disease, over 1 billion people globally are affected by mental and addictive disorders, which cause 19% of all years lived with disability worldwide (1). It is clear that such an extensive issue requires an urgent and committed public health effort. To that end, a range of population-level approaches have emerged, though the frameworks used and adopted vary widely.

## From Genome to Exposome

In recent years, a broad social agenda of psychiatric genetic research has emerged highlighting that genes account for a minority of our emotional and behavioral development, leaving the majority determined by social and physical environmental influences (2). Mental health must therefore be considered a dynamic state, whereby individual psychosocial development is influenced by multiple layers of intersecting social and environmental factors.

This starts in the womb with the mental and physical health status of mothers during pregnancy impacting the developing fetus. The very early years are greatly affected by parental bonding and the home environment. Thereafter, factors such as neglect or abuse in childhood; unemployment, poverty, and physical health problems in adulthood; and levels of social and community connectedness in later life all have a part to play in influencing an individual's ability and opportunity to access mental health protection, such as can be found in positive relationships, quality employment, and healthy living conditions. This picture is often complicated by the role of issues such as intergenerational trauma and the far-reaching and sustained impact of adversity in childhood, which suggests that upstream interventions are critical when taking a public mental health approach (3).

**Figure 1:**
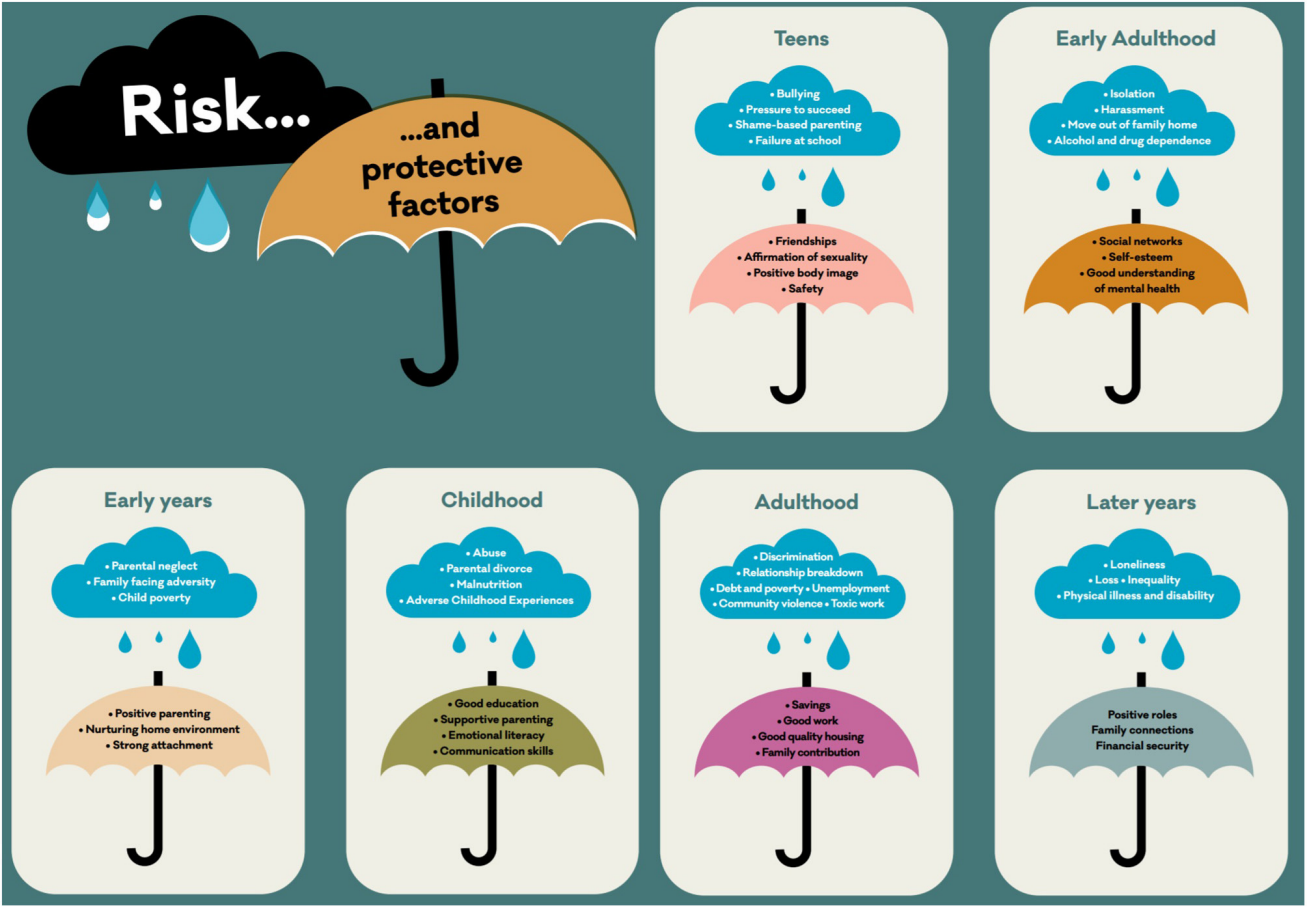
Visualization of some indicative risk and protective factors across various stages of the course of life. (c) Kousoulis, Mental Health Foundation.

These factors (an indicative range of which is presented in Figure 1) can and often do have a cumulative and intersecting effect across the course of life. Exposure to adverse experiences can be particularly common and clustered within some families and communities as well as specific settings, such as schools and workplaces, that are already experiencing other destabilizing factors. Hence, in many ways one's postal code is more important than one's genetic code (4), and the term “exposome” has been proposed as a new paradigm to encompass the totality of human environmental (meaning all non-genetic) exposures from conception onward, complementing the genome, and impacting human health (2). It is therefore crucial that if mental health problems are to be prevented, the social, economic, health, and ecological environment in which they are developing needs to be addressed (5). Understanding how these factors influence risk and how this can be mitigated is vital, as is communicating them in an accessible, visual way.

## From Public Health to Public Mental Health

Within public health there is indeed recognition that health is determined by a multi-dimensional and symbiotic relationship between the individual and the layers of their environment from the family home, through to the wider social and physical environment in which we live, including its cultural and political context. This describes a socio-ecological approach to health that has been advocated for almost three decades (6) and is still in line with the modern proposed definition of public health: “Public health is what we build together as a society when we shape our communities so everyone can achieve optimal health.” (7).

This public health view has much to offer within a mental health context where there can be a tendency to focus attention on the individual in isolation from the social and ecological conditions in which they exist. Hence, applying such an approach to mental health would make sense given the established knowledge behind the social determinants of mental ill-health (8). While ecological approaches have been proposed before in association with rural workforce and family-focused practice (9), the built environment (e.g., urban design and planning, green space access), and the social environment (e.g., prosocial spaces, civic participation, cultural resources) of cities lend themselves well to a population mental health approach (10). It is expected then that this socio-ecological factor could be more readily applied in urban settings, which include a more limited and specific built environment and better defined authority and resources (11).

In fact, more than half of the world's population currently lives in cities, and the trend for further urbanization is rapidly increasing (12). However, cities do not work for everyone. The bond between health and place is broken. Health systems treat acute illness, while urban systems promote chronic distress (13). To turn the tide on creating healthy urban places, we need to influence the culture, structure, and beliefs that drive priorities and values for healthy places by recognizing that humans need to live with meaning, including having a sense of purpose and of being of value and having a place where they belong.

**Figure 2:**
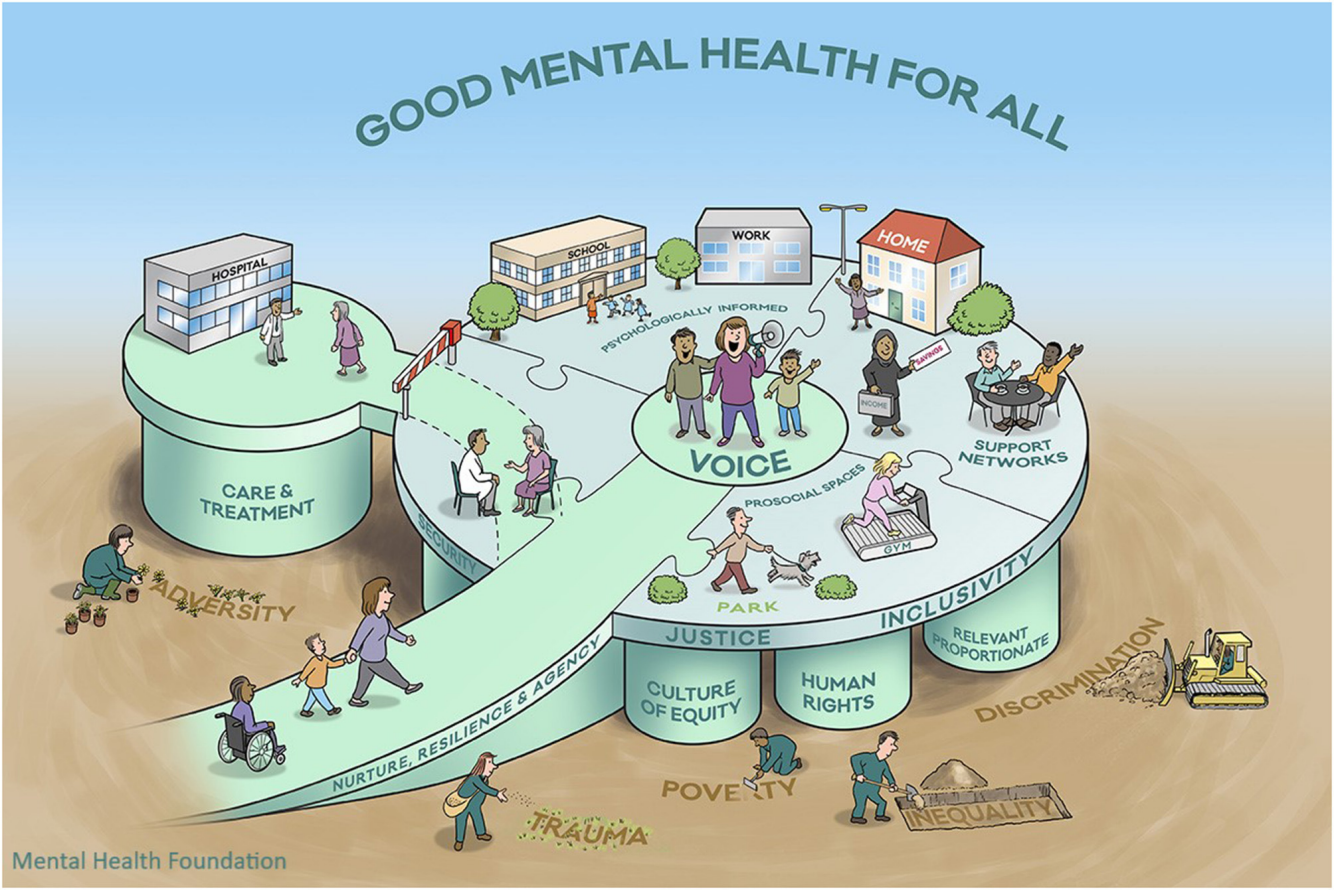
Visualization of a whole-city socio-ecological model for public mental health. (c) Kousoulis, Goldie, Mental Health Foundation.

To facilitate this conversation, we are proposing a new visualization for the traditional socio-ecological model approach to help frame urban public mental health programs (Figure 2).

## Discussion

In creating this new visualization for a socio-ecological model in public mental health, we have respected representation of the following evidence so that the visual image should:

(1) Look at both the individual person and that person's environment, family, and community, as well as the wider structures, culture, and beliefs, taking a universally proportionate and culturally relevant approach across the whole life course (14).
(2) Be relevant to the environments that impact human development. These classically include four systems (15):
  a) Microsystems: immediate social and physical surroundings (home, family, neighborhood, friendship groups);
  b) Mesosystems: wider systems within the environment (schools, health care);
  c) Exosystems: social, political and economic conditions (policy and legal environment including housing and welfare systems, cost, and standards of living);
  d) Macrosystems: beliefs and attitudes shared by members of society (including stigma and prejudice, views on social justice, equality, and inclusion).
(3) Apply a Health in All Policies (HiAP) approach, as there are limitations to funding, planning, and developing solutions in a health service silo (16). Health needs to be understood as a central factor in not only our individual success but that of society. This is even more important in the consideration of mental health, which has a powerful and pervasive influence on our ability to perform and be productive across a range of areas.
(4) Adopt a Whole Communities Approach. This acknowledges that mental health improvement interventions must operate across multiple system levels. Much of the improvement impact will be experienced not only within the health system through a reduction in more acute and long-term mental health and social care support services, but also across systems that rely on social capital (workplaces, schools), the public support infrastructure (health and welfare systems, housing, community/urban planning, and regeneration), and those parts of the system that are the endpoint for those whose distress has not been addressed at an earlier stage (criminal justice, homelessness) (17).
(5) Draw from Socio-Ecological Systems Theory (18), by acknowledging the central influence of beliefs and ideologies across society in systems development, while taking account of the interconnections and dependencies among community members.
(6) Acknowledge the way in which stigma and discrimination shape the cultural context of how mental health is viewed and valued and the level of effort society members are willing to expend to address the adversity and structural risk factors behind this (5).
(7) Position the voice of those with lived experience of mental health problems at the core of any decision-making process and ensure coproduction (working in equal partnership) is championed in designing services, campaigns, or prevention programs (5).

We invite a critique and conversation in using this strongly evidence-informed visualization when taking whole-city approaches and modern public health service design.

## Author Contributions

All authors participated in the conception, drafting, and editing of the manuscript and are accountable for the content of the work.

## Conflict of Interest

The authors declare that the research was conducted in the absence of any commercial or financial relationships that could be construed as a potential conflict of interest.
